# Extended clinical features associated with novel Glis3 mutation: a case report

**DOI:** 10.1186/s12902-017-0160-z

**Published:** 2017-03-02

**Authors:** K. A. Alghamdi, A. B. Alsaedi, A. Aljasser, A. Altawil, Naglaa M. Kamal

**Affiliations:** 1 King Abdullah Bin Abdulaziz University Hospital, Riyadh, Kingdom of Saudi Arabia; 2 Alhada Armed Forces Hospital, Taif, Kingdom of Saudi Arabia; 3 Prince Sultan Military Medical City, Riyadh, Kingdom of Saudi Arabia; 4 Pediatrics and Pediatric Hepatologist, Faculty of Medicine, Cairo University, Cairo, Egypt; 5 Pediatrics and Pediatric Hepatologist, Alhada Armed Forces Hospital, Taif, Kingdom of Saudi Arabia

**Keywords:** Clinical features, GLIS3 mutation, Abnormal genitalia, Saudi

## Abstract

**Background:**

Mutations in the GLI-similar 3 (GLIS3) gene encoding the transcription factor GLIS3 are a rare cause of neonatal diabetes and congenital hypothyroidism with 12 reported patients to date. Additional features, previously described, include congenital glaucoma, hepatic fibrosis, polycystic kidneys, developmental delay, facial dysmorphism, osteopenia, sensorineural deafness, choanal atresia, craniosynostosis and pancreatic exocrine insufficiency.

**Case presentation:**

We report a new case for consanguineous parents with homozygous novel mutation in GLIS3 gene who presented with neonatal diabetes mellitus, severe resistant congenital hypothyroidism, cholestatic liver disease, bilateral congenital glaucoma and facial dysmorphism. There were associated abnormalities in the external genitalia in form of bifid scrotum, bilateral undescended testicles, microphallus and scrotal hypospadias which might be a coincidental finding.

**Conclusions:**

We suggest that infants with neonatal diabetes associated with dysmorphism should be screened for GLIS3 gene mutations.

## Background

The Gli-similar family of Kruppel-like zinc finger proteins is comprised of three proteins, Glis1-3. Glis1 was first identified by a yeast-two-hybrid screening using the ligand-binding domain of the retinoic acid-related orphan receptor γ (RORγ) as bait [1]. Subsequently, two additional members of the family were identified that possessed high levels of homology with the zinc fingers of Glis1 and were termed Glis2 and Glis3 [2, 3]. The human GLIS3 gene is located on chromosome 9p24.2 and encodes a protein that is approximately 90 kD in size [4]. Mutations in GLIS3 (9p24.2, OMIM#610192) have been described in the literature as a rare cause of neonatal diabetes. GLI-similar 3 (GLIS3) is identified transcription factor containing five Kruppel-like zinc finger motifs [5]. GLIS3 expression occurs early in embryogenesis and is thought to play a critical role in the cellular regulation of development by functioning as a repressor or activator of transcription [5, 6]. In 2003, Taha et al described two siblings for consanguineous Saudi Arabian family had intrauterine growth retardation, neonatal diabetes and hypothyroidism, progressive hepatic fibrosis, renal cystic dysplasia, facial dysmorphism and congenital glaucoma [7]. In 2006, Senee et al performed Genome wide linkage analysis and sequencing of candidate genes on this family and they identified a homozygous frame shift mutation (c.1873dupC) in GLIS3 gene which is likely to result in transcript degradation by nonsense mediated decay [8]. These patients died from infection in infancy. Later on, 12 patients with mutations within the GLIS3 gene have presented with a wider phenotype consisting mainly of neonatal diabetes and congenital hypothyroidism, in addition to multiple features involving different organs. Dimitri et al described the genetic and clinical features of those 12 patients who had GLIS3 mutation [9]. The variation in the GLIS3 phenotype is attributed to the tissue expression of variable length transcripts derived from the 11 exon GLIS3 gene [10]. GLIS3 is expressed in a tissue-specific manner with the highest levels of expression observed in multiple tissues including testis, although genital abnormalities have not previously manifested in those patients. We describe a new case in which mutation in GLIS3 have resulted in severely affected patient with an extended phenotype including abnormalities in external genitalia that have not been previously described.

## Case presentation

Our patient is a male, who was born with a weight of 1.3 kg, length of 42 cm and head circumference of 29.4 cm after 36 weeks of gestation, to consanguineous Saudi parents (1^st^ degree cousins). He developed diabetes on day 2 after birth, requiring continuous intravenous insulin (0.03 IU/kg/h) and was subsequently treated with subcutaneous insulin (glargine once daily and Aspart as needed). Target blood glucoses have been difficult to achieve due to labile glucose level. His current daily dose of insulin is 0.4 IU/kg/day. Glycosylated haemoglobin was difficult to interpret due to persistently high levels of fetal haemoglobin.

On day 3 of life, hypothyroidism was identified. His cord thyroid stimulating hormone (TSH) level was extremely high. Serum TSH was 270μIU/L (normal 0.27–4.2) and free thyroxine (FT4) of 0.4pmol/L (normal 12–22). Thyroglobulin (TG) level was 427 ng/ml (normal 3.5–77). Maternal thyroid functions were normal. He was initially managed by oral Thyroxine20 mcg/kg per day of (25 mcg daily). Suppression of TSH proved difficult despite consistently normal FT4 measurements. Serum TSH concentration remains at above 100 μIU/L despite high dose of oral thyroxine (50 mcg/kg per day). Thyroid anatomy was normal on ultrasound scan while radioiodine scan was not done due to technical difficulties.

Patient developed early cholestasis. His total bilirubin was 289 umol/L (normal 2–193) with high conjugated bilirubin 103 umol/L (normal 0–5) and high gamma-glutamyl-transpeptidase 432 U/L (normal 8–61). His alanine aminotransferase and aspartate aminotransferase were normal. TORCH screen was negative. Ultrasound abdomen showed normal liver size and echogenicity. hepatobiliary scintigraphy showed patent biliary to bowel transit excluding biliary atresia with preserved hepatocyte function. Liver biopsy was refused by his parents but his liver function had improved gradually after starting of ursodeoxycholic acid orally.

Ophthalmic examination showed bilateral severe glaucoma so; he underwent goniotomy and trabeculotomy in early neonatal period. He has subtle dysmorphic features in form of depressed nasal bridge, pointed chin and ocular hypertelorism. Cranial and renal ultrasound scan were normal. Skeletal survey showed no skeletal abnormalities. He has normal hearing test.

His genital examination showed stretched phallus length of 1.8 cm with scrotal hypospadias, ventral chordae and bifid scrotum. Gonads were palpable in the inguinal canal. Karyotyping showed 46XY. Ultrasound of the pelvis and inguinal region showed no mullerian system, both testicles are in the distal inguinal region at the external ring with normal echogenicity and normal vascular perfusion.

Cortisol level was 395 nmol/L and he was maintaining normal electrolytes and serum glucose. Adrenocorticotropic hormone (ACTH) level was normal; 5.1pmol/L. 17-hydroxy-progesterone (17-OHP) was 2.3 nmol/L. Human chorionic gonadotropin (hCG) stimulation test was done at age of 5 months. Patient was given daily intramuscular 1500 IU/m2 of hCG for 3 days. Androstenedione, testosterone and dihydrotestosterone pre and post hCG stimulation were shown in Table 1. According to the laboratory results showed in Table 1, 5-alfa reductase deficiency, partial androgen insensitivity, 17-beta-hydroxysteroid dehydrogenase deficiency and congenital adrenal hyperplasia are unlikely to be the cause of genital abnormalities in our patient.

**Table 1:** Androstenedione, testosterone and dihydrotestosterone pre and post hCG stimulation

	Pre hCG stimulation	Post hCG stimulation
Androstenedione	16.8 ng/dl	43.2 ng/dl
Testosterone	20.2 ng/dl	52.5 ng/dl
DHT	2.4 ng/dl	5.4 ng/dl

*hCG* human chorionic gonadotropin, *DHT* dihydrotestosterone

## Genetic analysis

### Methods

Consent was obtained from his parents to perform genetic analysis. The 10 coding exons (exons2–11) of the GLIS3 gene on chromosome 9p24.2 (OMIM 610192) mutations were amplified by polymerase chain reaction (PCR) and sequenced directly. The resulting sequence data were compared with the reference sequence NM_001042413.

### Results

Detection of homozygous mutation c.23113_2314dupTC (p.Pro772Leufs*35) in the GLIS3 gene.

## Interpretation

Sequence analysis revealed a homozygous duplication of two nucleotides at position c.2313_2314 in exon 9 of the GLIS3 gene (c.2313_2314dupTC). This leads to a frameshift, resulting in premature termination codon (p.Pro772leufs*35) and subsequently in degradation of the mRNA (nonsense-mediated decay) or in a truncation of the protein. To best of our knowledge, this mutation has not been described in the literature so far and can be regarded pathogenic. Furthermore, the silent variant c.176G > T (p.Arg589Arg) in the axon 5 of GLIS3 gene could be detected in the homozygous state. Analysis by different bioiformatic tools did not predict splicing. This variant can most likely be regarded a pathogenic. The result was confirmed by sequencing of an independent PCR product. Although sequencing analysis cannot exclude a large heterozygous deletion in the GLIS3 gene in trans to c.2313_2314dupTC (i.e hemizygosity), homozygosity for c.2313_2314dupTC is most likely. Both homozygosity and hemizygosity for c.2313_2314dupTC in the GLIS3 gene would be compatible with the clinical diagnosis of our patient. In order to distinguish between homozygosity and compound heterozygosity for c.2313_2314dupTC and large deletion comprising this part on the allele we did analysis for the mutation in both parents through the same methods in the same genetic laboratory. Sequence analysis for both parents revealed a heterozygous duplication of two nucleotides at position c.2313_2314 in exon 9 of the GLIS3 gene (c.2313_2314dupTC), resulting in premature termination codon (p.Pro772leufs*35) and subsequently in degradation of the mRNA (nonsense-mediated decay) or in a truncation of the protein. Furthermore, the silent variant c.176G > T (p.Arg589Arg) in the axon 5 of GLIS3 gene was detected in the heterozygous state. Analysis by different bioiformatic tools did not predict significant scores for aberrant splicing. This variant can most likely be regarded a pathogenic.

## Discussion

We herein describe a new patient who has a novel homozygous mutation of GLIS3 gene with duplication of two nucleotides at position c.2313_2314 in exon 9 of the GLIS3 gene (c.2313_2314dupTC).

In agreement with all previous reports of GLIS3 mutations, our patient had neonatal diabetes, congenital hypothyroidism, congenital glaucoma, hepatic cholestasis, intrauterine growth retardation, developmental delay and characteristic facial dysmorphism but there was absent renal, hearing and skeletal involvement.

In this patient, we have described congenital hypothyroidism that has not responded to conventional treatment. Eleven out of twelve patients described with GLIS3 mutation, had high TSH and low FT4. In most cases, patients have not responded to conventional treatment and maintained elevated levels of TSH despite normalization of T4. However, abnormalities in thyroid anatomy and/or T4 uptake are not sufficient to explain this [10]. Three patients described by Senee et al responded to daily T4 treatment, but subsequent TSH levels were not reported. Thyroid ultrasound and scintigraphy results also suggested athyreosis or hypoplasia with absent radioiodine uptake [8]. Patients described by Dimitri and Taha had high daily T4 requirements with persistently elevated TSH and increased TG levels despite normal thyroid anatomy which are similar to our patient [7, 11]. One patient of *GLIS3* mutation with a deletion of exons 1–2, hypothyroidism was identified on day four of life with TSH levels >150μIU/l and T4 at 4.3pmol/l. The patient was treated with 20 μg/kg of T4 daily with sufficient TSH suppression. However, at 2 months of age, TSH levels exceeded 150mIU/l and remained high despite and increased dose of 75 μg/kg daily [11].

Although it is clear that almost all patients with a GLIS3 mutation present with thyroid dysfunction, the absence of consistent pathological features in patients makes it difficult to ascertain a unifying causative mechanism. It is possible that the markedly elevated levels of TSH observed in our patient and those described previously, combined with the variation in thyroid anatomy are a result of partial to complete TSH resistance. However, this does not explain the variable reduction in TSH, on occasions, values within the normal range following initial T4 supplementation, and the need thereafter to administer T4 three times daily to normalize free T4 [7].

*GLIS3* interacts with key regulatory genes in pancreatic embryogenesis including *ONECUT1* and *NEUROGENIN3* (*NEUROG3*) [11–13]. *GLIS3* expression also persists beyond the embryonic period promoting beta cell proliferation and regulating insulin gene expression through binding to GLI-RE on the *INS* gene [14, 15]. Therefore, the variation in insulin sensitivity between patients with GLIS3 mutations may relate to the impact of the mutation on the nuclear localization, GLI-binding element activity, transactivation, pancreatic development, subsequent cell proliferation, and remnant endogenous insulin production. In humans *GLIS3* has been identified as a susceptibility locus for the risk of type 1 and 2 diabetes [15, 16].

As previously reported, most of the patients who had *GLIS3* mutations presented with renal parenchymal disease, primarily renal cystic dysplasia [8]. However, some patients (including our patient) with mutations in *GLIS3* did not develop renal disease. The variable presentation of renal disease therefore may be related to the relative qualitative and quantitative expression of tissue transcripts and the encoded proteins in individual patients, or alternatively the variation in the expression of regulatory transcripts.

Our patient has abnormalities in the external genitalia that have not been previously described. We believe that this is probably a coincidental finding. Whether or not GLIS3 mutations would be the cause of these abnormalities needs extensive genetic testing and work up which are beyond the scope of our report which aims to report the recently discovered novel mutation of GLIS3. If any other reports are released in future literature this might raise the need for more work up of these patient but at the level of our report we can’t raise this suspicion as being a coincidental finding is much more logic specially with the base line relatively high incidence of these findings in male neo borns with 5 in 1000 and 1–5 in 100 newborns for scrotal hypospedius with bifid scrotum [17, 18] and undescended testis [19] respectively.

## Conclusions

In summary, we have described a patient with novel deletions in GLIS3, whose clinical phenotype includes more features than previously described and whose hypothyroidism was relatively resistant to conventional interventions as well as neonatal diabetes with labile blood glucose level. The extension of the phenotype, we have described, is probably coincidental.

## Abbreviations

17-OHP: 17-hydroxy-progesterone
ACTH: Adrenocorticotropic hormone
DHT: Dihydrotestosterone
FT4: Free thyroxine
GLIS3: GLI-similar 3
hCG: Human chorionic gonadotropin
PCR: Polymerase chain reaction
RORγ: Retinoic acid-related orphan receptor γ
TG: Thyroglobulin
TSH: Thyroid stimulating hormone
